# The relationship of light exposure to sleep outcomes among office workers. Part 1: Working in the office versus at home before and during the COVID-pandemic

**DOI:** 10.1177/14771535221136096

**Published:** 2022-12-15

**Authors:** MBC Aries, G Fischl, A Lowden, F Beute

**Affiliations:** a School of Engineering, Jönköping University, Jönköping, Sweden; b Stress Research Institute, Stockholm University, Stockholm, Sweden; c LightGreen Health, Rena, Norway

## Abstract

The relationship between everyday light exposure and sleep was studied for office workers. The study was conducted during the upswing of the COVID-19 pandemic, enabling a comparison between Office and Home Workdays. Fifteen full-time office employees were monitored for a period of 4–6 weeks. They wore a light-tracking device on their clothes and had a sleep tracker at home. Compared to an Office Workday, light exposure was lower in the afternoon and total sleep time was almost 5 minutes longer on a Home Workday. Sleep efficiency was the same on both workday types. A higher median illuminance level in the afternoon was significantly related to later sleep onset on an Office Workday. Higher median illuminance levels in the morning were related to earlier awakening. Counter to expectations, higher light levels in the evening were also related to earlier awakening. Everyday light exposure matters for sleep quality but may affect circadian functioning differently than the often more extreme light interventions employed in laboratory experiments. Moreover, differences in outcomes between Office and Home Workdays signal the need for further investigation to provide supportive light levels during workhours.

## 1. Introduction

A good night’s sleep gives the best start to the day. Sleep has been linked to mental and physical health aspects, ranging from better mood to lower overall mortality.^1–3^ Sleep is negatively affected by workload, work hours and perseverative cognition^4,5^ and personal factors such as having small children or not. Depending on its level, spectral distribution, timing and duration, exposure to light can affect human sleep and circadian functioning.^6,7^ In modern society, it is no longer (only) the sun that dictates the human rest-activity cycles.^8,9^ Social constraints (i.e. work schedules) and electric lighting available at any time affect sleep-wake rhythms in parallel. With an 8-hour working day, office workers spend about half of their time awake in office environments and, additionally, before and after work, considerable time at home. This activity pattern makes office and home lighting significant contributors to daily light exposure.^10^ Light levels in indoor environments are significantly lower (often <1000 lx) than exposure to daylight outdoors (often >1000 lx).^7,11,12^ Still, it is not only bright light exposure over 1000 lx that affects circadian functioning.^12,13^ Low to medium light levels have been reported to affect well-being and sleep.^6,14–16^ Especially in a Scandinavian setting, lighting at home usually has lower correlated colour temperatures than lighting used in commercial buildings. Traditionally this means that the lighting had a lower short-wavelength content. However, Cain *et al.*^17^ noted that homes with energy-efficient lamps had higher evening light exposure and consequently poorer sleep efficiency, potentially as energy-efficient fluorescent and LED lighting tends to double the amount of melanopic daylight-equivalent illuminance (mEDI) levels compared to incandescent light sources.

Light effects on sleep have been investigated in naturalistic, ambulatory studies looking at human light exposure and (self-reported) sleep quality, usually for 7 consecutive days.^18–22^ These studies looked at all-day light exposure, all-day short-wavelength spectral irradiance, morning/mid-day/evening exposure, and the proportion of bright light exposure, sometimes in different seasons. The studies found significant effects of daytime exposure on sleep, including earlier mid-sleep after higher levels of morning light exposure^20^ and more pronounced effects in winter,^18^ but not significant relations between mid-day exposure and sleep.^20^ Nevertheless, in some cases, light exposure was measured via a wearable worn on the wrist^20^ or upper arm.^22^ A recent systematic review on daily light exposure, sleep-wake rhythm, and mood reported limited and conflicting evidence for a positive relationship, largely due to the lack of intervention studies and the use of wrist worn light exposure devices.^23^

In the spring of 2020, the COVID-pandemic made several governments and public health authorities worldwide order or recommend employees to work from home, if possible. Office workers typically spend a substantial part of their waking hours in the office. This shift to working from home affected many aspects of the work environment, including light exposure. Lighting environments may differ between the office and the home, as lights in the home environments are usually more focused on creating a cosy atmosphere.^24^ Working from home may also induce behavioural shifts that can impact light exposure. First, workers may no longer need to commute from their home to work. This outdoor commute usually contributes considerably to the daily light exposure.^12,25^ In addition, with disappearing commute time, people tend to change their sleeping times.^26^ Third, a recent study has indicated that office workers spent more time behind a screen during the COVID-19 pandemic.^27^ Screen time can significantly contribute to light exposure; particularly, increased screen time in the evening can delay sleep onset.^28,29^ Xiao *et al.*^30^ showed that, during Home Workdays, overall mental and physical well-being decreased due to, for example, the lack of physical exercise or communication with co-workers, changed food intake, adjusted work hours, or the workstation set-up. In addition to social and behavioural changes, prolonged sedentary activity due to continuous online meetings at improvised desks (i.e. kitchen table) lacking proper electric lighting and daylight access can undoubtedly affect an employee’s light exposure.

The effects of light exposure on sleep have been proposed to depend not only on the light amount, spectral composition and timing of the light but also on, for example, season,^31^ duration,^32^ or light history.^33^ Therefore, in the current study, office worker’s naturally occurring light exposure and sleep were monitored for multiple consecutive weeks, both during and outside working hours, using a 24-hour ambulatory assessment during one season (spring). The present study started when the upswing of the COVID-19 pandemic was lurking, but no measures were announced or instituted yet. During the study, COVID-measures were implemented, and employees were asked to work from home as much as possible.

The present study captured potential differences in sleep quality/patterns and light exposure between working from home and working in the office. The effects of light exposure on sleep outcomes were investigated at three times: morning, afternoon and evening. Light variables under study were (vertical) illuminance level and red (R), green (G) and blue (B) values. Due to the high unreliability of the RGB values, these results were not considered. It was hypothesised that:

1. An increase in light exposure in the morning results in higher sleep efficiency and earlier sleep onset and offset,
2. An increase in light exposure in the evening results in lower sleep efficiency and later sleep onset and offset (i.e. an opposite, but less-pronounced pattern from the morning exposure),
3. An increase in afternoon light exposure does not influence sleep outcomes since it is outside the critical period for sleep advance (between 06.00 and noon) and sleep delay (midnight and 06.00), and
4. Light exposure was lower on Home Workdays than on Office Workdays, which would negatively affect sleep outcomes.

## 2. Method

In an ambulatory field study, 15 participants worked from home more often during the second half of the study, which monitored their personal light exposure and sleep quality for 4–6 weeks.

### 2.1 Participants

Office employees of two different companies and within different buildings in Sweden (~57°N, 14°E) were invited to participate. In total, 15 participants (seven females, age between 29 and 63 years, *M* = 43.3 ± 10.4) were included in this study. If a participant was pregnant, used sleep medication, was professionally diagnosed with a sleep disorder, worked less than 4 days per week, or had children younger than 1 year, he/she was excluded from participation. An online questionnaire was administered before and after the experiment. It included questions on demographics (age, gender, weight, height, household arrangement), commuting behaviour, regular working hours, sports and leisure activities, and long-term subjective sleep quality (Pittsburgh Sleep Quality Index^34^).

### 2.2 Materials

Personal light exposure was measured with a wearable light detector (LYS Button by LYS Technologies LTD™) at a 15-second interval. The light detector was worn as close to the eyes as possible during the daytime and placed on the bedside table at night. The detector measures illuminance (in lux) as well as (R), (G) and (B) 16-bit values. Validation is performed on request of the manufacturer itself, but the results are not published. All 15 light detectors were checked and calibrated prior to the study, both for indoor and outdoor light conditions, against two Hagner EC1 illuminance meters (calibrated by B. Hagner AB in February 2019 with limited use after; calibration accuracy ±3%). A correction factor was applied to all detectors. Differences for indoor conditions were between 0 and 5%, while outdoor values showed much higher differences. Thus, for typical indoor conditions (up to 5000 lx), the light detector performs appropriately. Additionally, the light trackers were investigated for their performance regarding spectral ranges for ‘R’, ‘G’ and ‘B’. However, the sensitivity curves for the B and G channels had a significant overlap, and hence the RGB values were not further considered in the study. Sleep variables were measured with a non-contact sleep tracking device (SleepScore Max by SleepScore Labs™) based on sonar and radiofrequency. The device analyses sleep quality entirely without contact with the participant as it uses bio-motion sensor technology, like echolocation, to track breathing and body movement. Validation is performed on request of the manufacturer itself by independent institutes and the results are published in peer-reviewed scientific journals.^35,36^ For example, Schade *et al.*^36^ compared the device against polysomnography and showed that the devices typically had a high sleep-detecting sensitivity (⩾95%) but poor wake-detecting specificity (⩽40%).

All participants received a 4G-equipped mobile phone (iPhone 6) with a charger containing data collection apps. The light tracker used the regular app (without data reveal as it went via a paid service). The sleep tracker used a special research edition of the app to prevent data reveal to the participants (‘R’ version).

The study was executed in two offices with open-plan and single office spaces in Fagerhult and Jönköping, Sweden. Building one is equipped with suspended direct/indirect luminaires (Fagerhult Notor 78; 50/50 or Fagerhult Notor Skywalker, 40/60) above each desk. The other building has ceiling-mounted direct luminaires (Fagerhult Multilume Slim) in the open-plan offices and suspended direct/indirect luminaires (Fagherhult Itza Delta, 50/50) in the single rooms. The lighting of both office buildings was connected to presence sensors. Daylight can enter the room via the windows unless this is prevented using Venetian blinds or screens. The daylight contribution in the office per orientation was logged every 5 minutes with an illuminance logger (YoYo 2YL-M61-4M/2YL-M62-4M by Grant Instruments) horizontally placed at the windowsill (nine loggers total). The vertical illuminance at each desk was logged using an illuminance logger (HOBO MX2202 by Onset Computer Corporation), hanging at the desk divider at eye height. Presence at the desk was registered using a wireless occupancy desk sensor and logger (iotspot Hub).

### 2.3 Procedure

The data collection took place both in the office and at home. Participants were followed for at least 4 weeks during late winter (between February 27th and April 6th). Prior to the experiment, for both companies separately, the experiment’s general set-up and procedure were explained. The participants were aware of partaking in an experiment related to lighting and sleep quality but unaware of the exact study purpose. All participants got a unique ID code to ensure confidentiality of their identity and gave their written consent for participation and data collection. The study protocol was approved by the Swedish Ethical Review Authority (no. 2019-05453).

### 2.4 Data analysis

Given the nested nature of the data, hierarchical linear models were used to test relationships between light exposure and sleep outcomes, with responses nested within individuals as well as within days. Separate models were run for the morning (06.00–11.00 hours), afternoon (12.00–17.00 hours) and evening (18.00–23.00 hours). Three sleep variables were included as outcome variables: sleep efficiency (ratio of wake time after sleep onset and total sleep time), sleep onset and sleep offset. Three different models were run: one Overall model looking at all workdays together and two models looking at Home Workdays and Office Workdays separately.

Median illuminance level per hour was added as a predictor. Data from the occupancy sensors were used to determine whether participants were in the office (>10% presence) or not (<10% presence). In this data, no distinction could be made between working at home, working outside the office the entire day (e.g. visiting clients even though unlikely during the pandemic), and days off on a weekday.

Only hours for which at least 40% of the data was above a threshold of 10 lx were included. As the illuminance values ranged between very low and very high peak values (e.g. between 10 and 29,825 lx), all values were log-transformed. Within the study period, the clock was set to summertime (March 29th). Therefore, data from 3 days after daylight saving time were deleted from the analyses.

## 3. Results

Several indicators for sleep and light exposure were monitored for office employees for at least 4 weeks during and after work time. As participants were recruited from two different companies, before starting the main analyses, it was tested whether outcome variables or light exposure differed between the two participant groups. They did not differ on any of the variables (all *p* > 0.155).

A total of 5550 valid hourly light measurements were included in the analyses, ranging from 90 to 452 measurements per participant, with a mean of 330 measurements. Missing data points could be due to user behaviour (e.g. going outdoors with a coat over the light sensor or forgetting to synchronise the device) or technical issues (e.g. no connection). As the participants had to switch on the sleep tracking device each night manually, there were some missing sleep data. A total of 355 sleep episodes were recorded, ranging between 13 and 30 per participant, with a mean of 24 measurements.

The percentage Office Workdays per person ranged from 20% to 88%, with an average of 0.49% (SD = 0.28). The percentage of Office Workdays was highest during the second week of the study, and lowest during the last week of the study (week 1: 64%, week 2: 68%, week 3: 45%, week 4: 36%, week 5: 33%).

**Figure 1:**
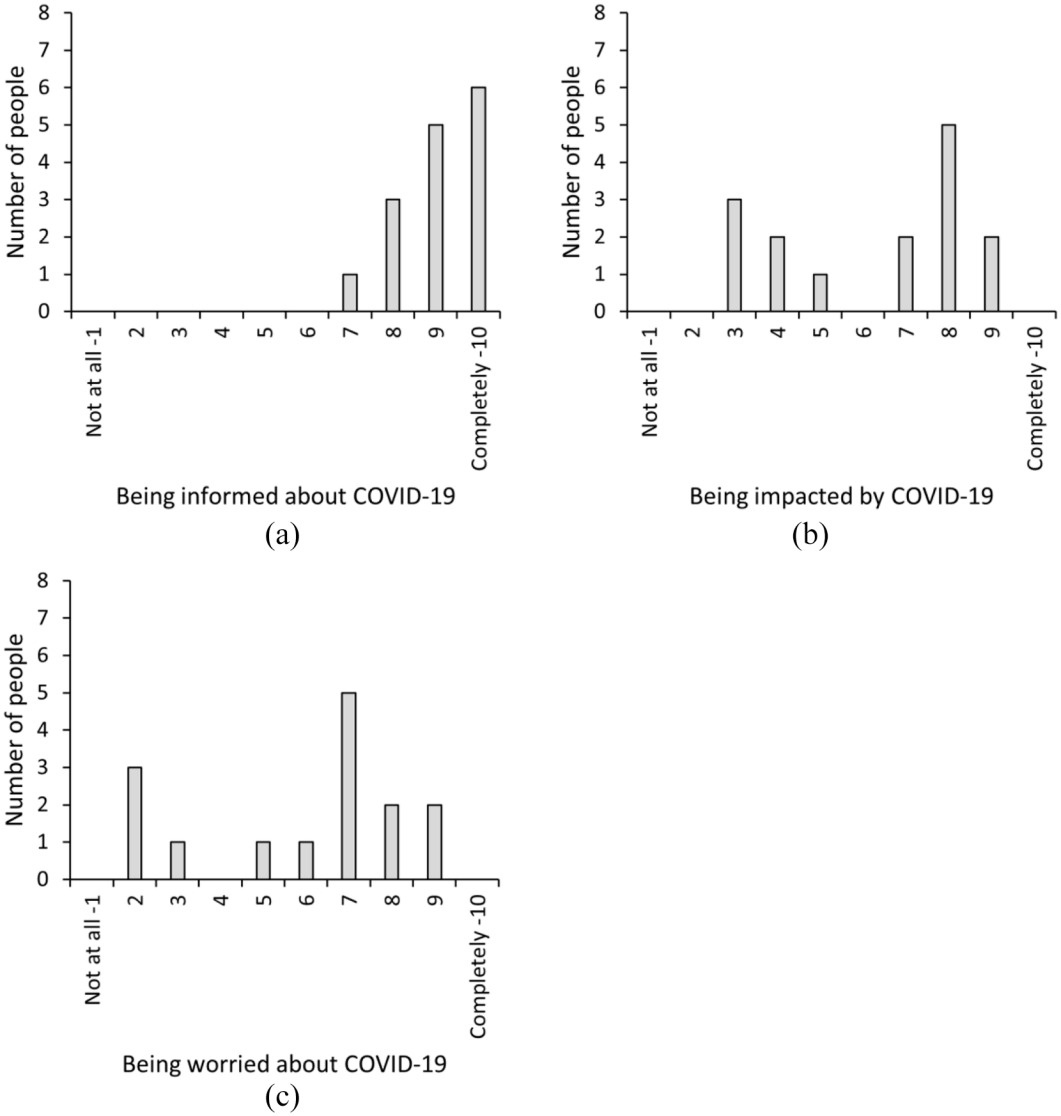
Outcomes of the COVID-19 pandemic control questions: being informed (a), impacted (b) and worried (c)

After the study was finished, three questions were asked to measure how the COVID-19 pandemic had influenced the participants. These questions concerned to what degree they felt informed about COVID-19, whether COVID-19 had impacted their lives, and how much they ruminated about COVID-19, see Figure 1 for the answers. No significant relation was found between these COVID-19 related questions and any sleep outcomes (all *p* > 0.293). In addition, the correlation between how much people worried about COVID-19 and the number of home working days was nonsignificant (*p* = 0.687).

### 3.1 Sleep outcomes

**Figure 2:**
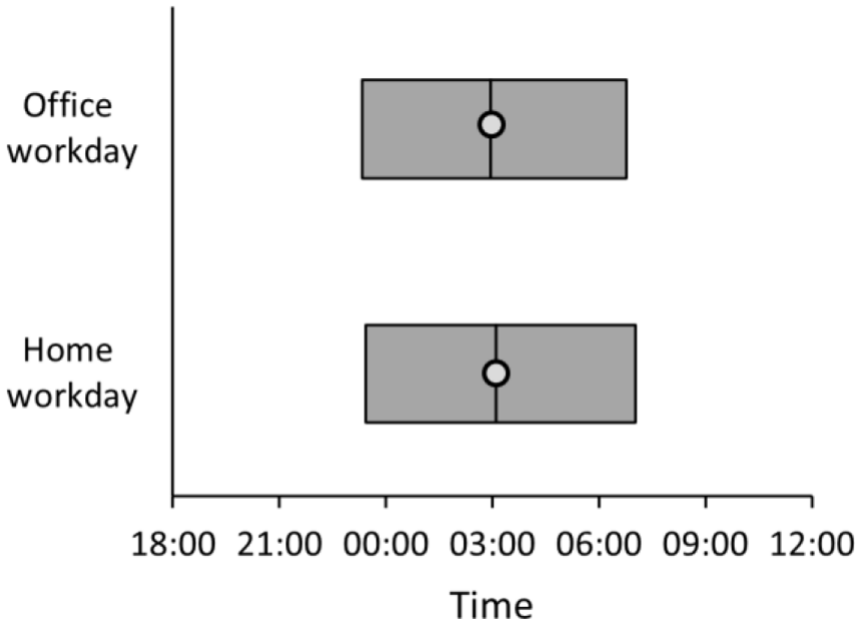
Sleep onset, midpoint sleep (circle), and sleep offset on Office Workdays and Home Workdays

Data were recorded for 195 workdays ‘at the office (Office Workday)’ and 108 workdays ‘not at the office (Home Workday)’. The data recorded for 105 ‘Weekend’ days were used in Beute *et al.*^37^ Sleep parameters on a Home Workday were compared to Office Workdays; see Table 1 for an overview of sleep outcomes for each type of day. Compared to an Office Workday, on Home Workdays, sleep onset was 6 minutes later, midpoint sleep was 9 minutes later, and sleep offset was 16 minutes later. Total sleep time was approximately 5 minutes longer on a Home Workday than an Office Workday and sleep efficiency was the same on Office Workdays and Home Workdays. See Figure 2 for an overview of the sleep times.

**Table 1:** Overview of the estimated marginal means (EMMs) with standard errors (SEs) for sleep outcomes for an Office Workday and a Home Workday

	EMM (SE) sleep outcomes	
	Office Workday	Home Workday
Sleep onset	23.20 (10.3 minutes)	23.26 (10.4 minutes)*
Sleep offset	06.46 (10.9 minutes)	07.02 (10.9 minutes)*
Midpoint sleep	02.57 (9.5 minutes)	03.06 (9.5 minutes)*
Total sleep time	395 minutes (11.36 minutes)	400 minutes (11.41 minutes)*
Sleep efficiency	89.44% (1.55)	89.80% (1.55)

* *p* < 0.05.

### 3.2 Personal light exposure

**Figure 3:**
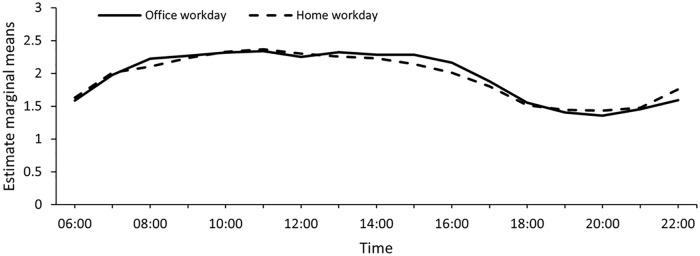
Mean values for the log-transformed median illuminance levels across an Office Workday and a Home Workday. The hours between 23.00–5.00 were excluded due to a low number of observations per type of day (< 20)

Light exposure differed between the two different workdays; see Table 2. Compared to an Office Workday, light exposure was lower in the afternoon on a Home Workday. Figure 3 displays the median light exposure across the day (median log-transformed illuminance). Afternoon light exposure was positively skewed in terms of light level, and results for the afternoon need to be interpreted with caution.

**Table 2:** Overview of EMMs with SEs for illuminance levels (log-transformed) for the 2 workday types for the three different hierarchical linear models (HLM) models

HLM model	EMM (SE) illuminance levels	
	Office Workday	Home Workday
Morning	2.19 (0.03)	2.14 (0.04)
Afternoon	2.23 (0.04)*	2.10 (0.04)*
Evening	1.48 (0.04)	1.49 (0.05)

* *p* < 0.05.

### 3.3 Relation light exposure and sleep outcomes

Table 3 shows the outcomes of the three different models for median illuminance level. Sleep offset on a subsequent day was earlier with a higher median illuminance level in the morning for both the Overall model and the Office Workday model. No effect of morning median light level on sleep offset was found for the Home Workday. In the afternoon, a higher median illuminance level was related to later sleep onset, but only for the Office Workday model. In the evening, the median illuminance level was negatively associated with sleep offset for the Overall model, with later sleep offset after being exposed to higher evening light levels. Additionally, median illuminance level was significantly related with sleep offset for an Office Workday, again with later sleep offset after being exposed to higher light levels. On a Home Workday, this relation did not reach significance.

**Table 3:** The unstandardised estimates (EST) with SEs, 95% confidence intervals (CIs) (lower (LCI) and upper confidence interval (UCI)), and intra class correlations (ICC) of the sleep outcomes for the three models for light exposure measured by the median illuminance level

	Sleep efficiency				Sleep onset				Sleep offset			
	EST (SE)	LCI	UCI	ICC	EST (SE)	LCI	UCI	ICC	EST (SE)	LCI	UCI	ICC
Morning												
Overall	0.54 (0.37)	−0.18	1.26	0.64	−0.96 (2.90)	−6.65	4.73	0.59	−6.01 (1.78)**	−9.50	−2.52	0.85
Office	0.81 (0.49)	−0.16	1.78	0.63	0.26 (3.17)	−5.95	6.47	0.65	−4.20 (2.04)*	−8.20	−0.20	0.87
Home	0.32 (0.45)	−0.56	1.20	0.78	1.50 (2.29)	−2.99	6.00	0.97	−2.82 (2.31)	−7.35	1.70	0.95
Afternoon												
Overall	0.24 (0.41)	−0.55	1.04	0.58	6.02 (3.17)	−0.19	12.23	0.55	−1.14 (1.80)	−4.68	2.93	0.85
Office	0.66 (0.55)	−0.40	1.74	0.57	14.96 (3.37)**	8.36	21.56	0.59	1.05 (2.01)	−2.90	5.00	0.88
Home	−0.23 (0.50)	−1.21	0.74	0.77	−0.11 (2.14)	−4.31	4.09	0.97	1.24 (2.23)	−3.14	5.61	0.94
Evening												
Overall	−0.05 (0.63)	−1.29	1.19	0.54	−9.17 (4.90)	−18.77	0.42	0.54	−7.82 (2.55)*	−12.82	−2.82	0.86
Office	−0.33 (0.82)	−1.93	1.26	0.54	−6.95 (4.37)	−15.51	1.51	0.63	−6.26 (2.88)*	−11.91	−0.62	0.87
Home	0.53 (0.76)	−0.94	2.02	0.70	−3.72 (3.35)	−10.30	2.85	0.97	−1.74 (2.09)	−5.84	2.37	0.98

* *p* < 0.05. **p < 0.001.

## 4. Discussion

In an ambulatory field study, office workers’ light exposure and sleep quality data were collected. The study captured personal light exposure from waking up in the morning to going to sleep at night during the start of the COVID-19 pandemic.

Due to the COVID-19 pandemic, participants worked from home more than usual as the study progressed. In Sweden, there was no strict lockdown, and therefore, social constraints on sleep times during Home Workdays might differ less from Office Workdays than in other countries with (stricter) lockdowns.^38^ In addition, not all participants were requested to work from home daily. Still, participants got out of bed, on average, 16 minutes later on Office Workdays than on Home Workdays and went to bed, on average, 6 minutes later. They also slept around 5 minutes longer, which is similar to what is reported in countries with a lockdown.^39^ No differences in sleep efficiency were found between Office and Home Workdays.

Three different models investigated the relationship between light exposure and sleep outcomes. Previous studies have indicated that home lighting often has lower intensities than office lighting.^19,40^ Nevertheless, results of a 3-week study investigating evening light levels demonstrated that self-chosen home light levels before habitual bedtime can increase circadian misalignment.^41^ even though there is often a wide range of individual responses.^17^ In the current field study, only lower afternoon light exposure was reported on a Home Workday compared to an Office Workday. Differences in light exposure might have been more pronounced if the effects of the lockdown had been studied during the mid-winter season.

A recent overview by Vetter *et al.*^42^ showed that bright light exposure in the morning phase advances sleep on a consecutive night and bright light exposure in the second part of the biological day phase delays sleep on a consecutive night. The outcomes of the present study only partly followed this expected pattern. In line with expectations, higher levels of light in the morning were related to an earlier offset (waking up) the day after for the Overall and the Office Workday model. However, counter to expectations, higher evening light exposure was related to an earlier offset in the Overall and Office Workday models. No significant relationships were found between morning and evening light exposure at a Home Workday and sleep outcomes. Potentially, these differences are due to lower statistical power as there were fewer Home Workdays in the sample than Office Workdays even though the model coefficients did point in the same direction.

The counterintuitive outcomes for the evening may be due to several factors. Previous research has pointed at a large variability in sensitivity to evening exposure^14,17^ and the importance of light history^33,43–45^; for instance, exposure to blue-enriched light in the morning or bright daytime light exposure counteracting higher light levels in the evening. In other words, effects of evening exposure may depend on how much light a person received in the morning (and when and with which composition). Phase-delaying effects of bright or blue-enriched electric light in the evening are often found in laboratory studies. Laboratory studies may differ in timing (with night-time light exposure as opposed to evening light exposure), duration (longer duration to higher light levels), and intensity/composition (using, for example, very bright light exposure and blue-enriched light). The test conditions may differ from the naturally occurring light levels in the present study. Potentially, the differences in light exposure in terms of vertical illuminance level in the present study are of a much more subtle nature than in laboratory experiments and therefore resulted in different outcomes. Alternatively, differences in outcomes compared to earlier studies could be due to the extended investigation time (4–6 weeks rather than 7 days). Detrimental effects of light exposure mostly appear later when participants had often already gone to bed on workdays. In addition, the results show that on both workday types, there was an increase in illuminance level after 20.00. Previous research has found that participants usually spend the last hour before going to bed watching television.^40^ An increased exposure might be explained by people continuing to work in the evening (with increased levels of electric lighting). Additionally, research has indicated that people spend more time behind a screen during the COVID-19 pandemic.^27^

No significant relationship was found with the sleep outcomes on the Home Workday or the Overall model for afternoon light exposure. However, for the Office Workday, a higher median illuminance level was found related to a later sleep onset. This effect only occurred for the Office Workday. As a significantly higher light exposure on the Office Workday compared to the Home Workday was found, and the direction of the (nonsignificant) relationship between afternoon light exposure and sleep outcomes for the other two models were in the opposite direction, it seems plausible that these variations were due to differences in light environments. However, as light exposure was positively skewed in the afternoon, this result needs to be corroborated in future research. Research often focuses on morning and evening light exposure; therefore, less is known about the effects of afternoon exposure to light on sleep. Other studies have not found a relationship between an afternoon or mid-day light exposure and sleep outcomes.^20,46,47^ The absence of a significant relationship between sleep quality/duration and light exposure in the morning or afternoon can possibly be due to the use of subjective sleep assessment tools (i.e. sleep diary^48^) rather than an objective instrument.

The applied light detector offered RGB-values measurement, which could have given – if sufficiently distinctive – an indication regarding the light exposure being,that is more short-wavelength enriched or deprived, even though a previous study reported a high correlation between RGB clusters and illuminance level.^19^ Nevertheless, daylight varies in spectral power distribution over the day, especially when comparing twilight versus midday compositions. This calls for studying more subtle daily variations in spectral power composition and their relationship with everyday light effects and sleep outcomes. Especially since it is not only the short wavelengths of light that deserve attention but also other wavelengths of the visual spectrum in daily life. At present, only a few studies investigated effects of longer wavelengths of light, often only as a control or placebo condition.

Several factors pose limitations to the present study. First, technical failures of the light detector and omissions of the participants switching on the sleep device led to missing data. Furthermore, as participants were instructed to wear the light tracker on their clothes, outdoor light exposure was not (correctly) captured when a coat blocked the sensor. Third, the study was not explicitly designed to capture differences between home and Office Workdays but caught them inadvertently. Differences in daily routines (e.g. not traveling to work) may have affected light exposure. In addition, even though participants occasionally worked from home in the beginning of the study, the frequency was higher during the end of the study. Small differences in light exposure due to changes in daylight hours may have affected the outcomes. Altered daily activities and mood changes or rumination levels due to the COVID-19 pandemic may have affected sleep during the experiment, besides light exposure. Last, age differences between participants may have led to differences in sleep outcomes and circadian effects of light, as older adults do not sleep as well as younger adults. For example, older participants (57–74 years) in the study of Münch *et al.*^49^ felt significantly sleepier and reported more sleep at circadian times corresponding to the late afternoon and evening (16.00–22.00 hours) compared to younger ones (20–31 years). As a result of ageing, some older people have difficulties staying awake during evening hours due to a weakening circadian wake signal. Fortunately, this is not the case for teenagers and young adults.^50^

The present study found that everyday light environments matter for sleep outcomes. In addition, differences between office light environments and home light environments may influence how employees sleep on an Office versus Home Workday but needs further investigation especially for the afternoon. Everyday light exposure appeared to affect sleep differently from results found in laboratory, especially with regards to evening light exposure. Potentially, the more subtle daily fluctuations in light exposure in everyday life, as opposed to the more extreme light manipulations in laboratory studies, may result in different relationships between light exposure and sleep.
